# Fracture Strain of Al–Si-Coated Press-Hardened Steels under Plane-Strain Bending

**DOI:** 10.3390/ma15207345

**Published:** 2022-10-20

**Authors:** Zeran Hou, Wei Song, Hongliang Yi, Jianfeng Wang, Junying Min

**Affiliations:** 1 Postdoctoral Station of Mechanical Engineering, Tongji University, Shanghai 201804, China; 2 School of Mechanical Engineering, Tongji University, Shanghai 201804, China; 3 Product Engineering Department of Nanjing Iveco Automobile Co., Ltd., Nanjing 210028, China; 4 State Key Laboratory of Rolling and Automation, Northeastern University, Shenyang 110167, China; 5 China Science Lab, General Motors Global Research & Development, Shanghai 201206, China

**Keywords:** fracture strain, press-hardened steel, bendability, plane-strain bending, Al–Si coating

## Abstract

Press-hardened steel (PHS) is widely applied to fabricate vehicle body structures for attaining mass reduction and fuel economy without sacrificing occupant safety. The VDA bendability test is often used to characterize the fracture resistance of PHS under plane-strain bending conditions. As lightweighting continues to be a design imperative in the automotive industry, it is desirable to develop and adopt more press-hardened components with higher fracture resistance. In this work, four Al–Si-coated 22MnB5 steels with various initial thicknesses and coating weights were studied. A newly developed methodology was used to calculate the fracture limit strain under plane-strain bending. The results indicate that although the four investigated 22MnB5 steels exhibit similar tensile properties under uniaxial tension, their bending performance per the VDA 238-100 standard differs significantly. The PHS with a low coating weight possesses a higher bending angle and, hence, a larger fracture limit strain. Meanwhile, the peak bending force can be 10% higher than the PHS with a standard coating weight at the same sheet thickness. Therefore, it is expected that PHS with higher fracture strain will have the potential for lightweighting due to its enhanced resistance to fracture and higher energy absorption capability.

## 1. Introduction

Weight reduction can be achieved with the use of high-strength materials, resulting in energy conservation and reduced emissions [1]. Due to their high strength and matured component manufacturing technology, press-hardened steels (PHSs) are widely used in automotive body structural components such as B-pillars, door intrusion beams, and bumper beams [2,3,4]. In recent years, new developments related to PHS are mainly focused on the following three areas [5]: The first route is to add Nb, V, and other microalloying elements to improve PHS for higher strength and better toughness. The second is the integration of the quenching and partitioning step with the hot stamping process to increase the tensile ductility of PHS [6]. Finally, the third is based on the surface protection of PHS, by either developing new Al–Si coating technology to increase the bending fracture resistance of the press-hardened component [7], or enhancing the surface oxidation resistance of the steel substrate [8]. It should be noted that although Zn or Zn–Fe coatings can provide some cathodic protection, their adoption by the automotive industry is rather limited, and their market share is far less than that of the Al–Si-coated PHSs due to the risk of liquid-metal-assisted cracking during hot stamping [9].

To fully exploit the possible applications of PHS, numerous works on collision and simulation have been carried out. For example, to study crack propagation behavior, the finite element method and the phase-field theory have been shown to be important tools, especially when introducing variables such as temperature and size factors [10,11,12]. While the method of simulation can provide references, the accuracy of the simulation also relies on accurate experimental data. Then, when experimental data are not directly available, simulation analysis may be a better option to guide the design. During a car collision, components will often fold and bend under similar deformation conditions as those of plane-strain bending. In a tight-radius bending operation, plane-strain bending corresponds to the lowest fracture resistance of sheet metals [13,14,15,16]. Therefore, the study of bendability provides critical information about the fracture resistance of press-hardened steels and press-hardened components. The VDA 238-100 three-point bending test procedure is widely used to assess the bendability of sheet metal under plane-strain conditions [17,18]. However, as the bending angle cannot be directly measured as bending strain at fracture, it cannot be transferred into a finite element model to predict fractures of structural components during crash events. When a thin sheet is subjected to bending deformation, the outer layer is under tension and experiences the largest deformation (i.e., stress and strain). Hence, the initial crack will typically first appear at the outer surface. For the ease of industrial application, the fracture event of the specimen can be recognized when the maximum load is reached during the instrumented three-point bending test. The through-thickness strain gradient of the bending condition suppresses the necking behavior, which is a common predictor of failure in tensile tests [13,19,20]. Hence, the strain of the outer layer of a bent sheet at the moment of the first crack’s appearance is treated as the fracture strain of the specimen under three-point bending conditions with a tight bending radius (which is also a plane-strain bending condition) [21]. For the same material thickness, a higher bending angle represents better ductility. It was reported [22] that the bending angle of the materials reflects the fracture strain under plane-strain bending. However, the fracture bending angle estimated by analytical formulae is thickness-dependent and is not transferable into fracture strain, which is usually applied to numerical models to predict the fracture of structural components. Clearly, simulations can be improved with more accurate strain data obtained via improved experimental methods. Thus, it is particularly important to establish a connection between the bending angle and fracture strain. Recently, a simple methodology was developed to confirm the fracture strain under three-point bending tests [21]. Interrupted bending tests and linear extrapolation were employed following the VDA 238-100 standard.

In the process of stamping transfer, it is inevitable that the surface of bare PHS will undergo oxidation and decarburization, which could increase the subsequent processing costs and pollute the environment. To solve the above problems, Al–Si coating technology was developed by ArcelorMittal [23]. Lawrence et al. [24] found that the bending angle of Al–Si-coated PHS with a pre-coated layer thickness of 30 μm decreased by 35% compared to bare PHS. Lawrence et al. [24] considered hydrogen-induced fracture to be the key factor. Recently, the patent of Yi et al. [25] studied the diffusion process of Al–Si coatings in depth and deduced that carbon enrichment between the coating and the substrate after alloying is the physical mechanism of the toughness reduction of the Al–Si-coated PHS. Without altering the coating composition, a PHS with an original coating of around 8~14 μm was developed. The thinner Al–Si-coated PHS showed an increase in the bending angle of over 20% compared with the PHS with a conventional Al–Si coating thickness under the same sheet thickness. It was shown [7] that thinner coatings obtained a better bending angle by eliminating the carbon enrichment between the matrix and the interdiffusion layer.

In this study, four Al–Si-coated 22MnB5 steels with different initial thicknesses and coating weights were chosen to characterize fracture strain under plane-strain bending conditions. Two of the selected 22MnB5 sheets were 1.4 mm thick, with pre-coated layer thicknesses of ~11 μm and ~33 μm, respectively. For ease of comparison, the sheets with a thinner pre-coated Al–Si layer (thickness of ~11 μm) are referred to as thin-coated sheets, while the sheets with a thicker pre-coated Al–Si layer (thickness of ~33 μm) are referred to as thick-coated sheets. The typical microstructure and mechanical properties of the thin- and thick-coated sheets were investigated in this study. The previously mentioned method [21] was employed to determine the fracture strain of the above steels.

## 2. Experimental Procedure

### 2.1. Materials and Characterization

Four Al–Si-coated 22MnB5 steels were investigated in this study. All of the cold-rolled Al–Si sheets were first provided by steelmakers at the size of 980 mm × 1200 mm (width × length) from production coils. The chemical compositions of these materials are given in Table 1. The materials with the pre-coated Al–Si layer thickness of ~11 μm were denoted as Thin_(1.4/1.8)_, while the materials with the pre-coated Al–Si layer thickness of ~33 μm were designated as Thick_(1.4/1.8)_. The actual sheet thicknesses of the Thin_1.4_, Thick_1.4_, Thin_1.8_, and Thick_1.8_ sheets were 1.39 mm, 1.41 mm, 1.84 mm, and 1.80 mm, respectively. Blanks with dimensions of 300 mm × 400 mm (with the longitudinal direction parallel to the sheet-rolling direction) were cut from the larger sheets using electrical discharge machining (EDM) and subsequently hot-stamped. Blanks that were 1.4 mm thick were heated to 930 °C at a heating rate of around 10 °C/s, and the total heating time (heating and soaking) in the furnace was 280 s. Meanwhile, blanks with a thickness of 1.8 mm were heated to 930 °C at a heating rate of around 10 °C/s, and the total heating time (heating and soaking) in the furnace was 340 s. The austenitized blanks were then transferred to a flat die and die-quenched in a 450-ton press with a holding time of 14 s. Then, the die-quenched blanks were taken out of the press with sheet temperatures below 100 °C. After hot stamping, all specimens were baked at 170 °C for 20 min to simulate the paint-baking process in industrial production lines.

**Figure 1:**
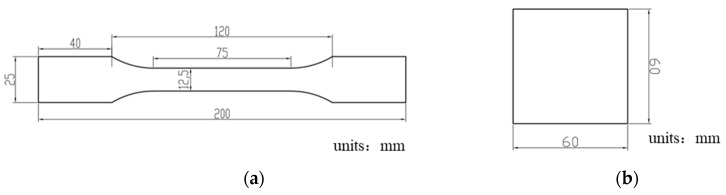

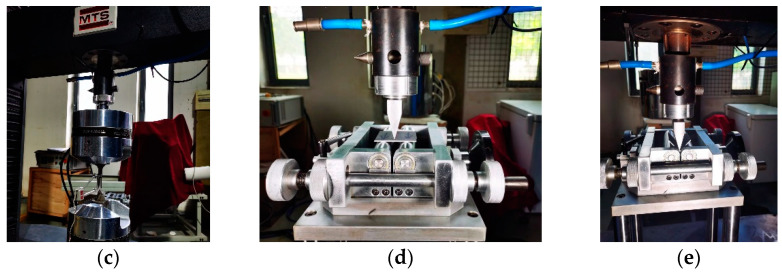
(**a**) Geometries of tensile specimens; (**b**) geometries of three-point bending specimens; (**c**) tensile testing machine with a 50 mm extensometer; (**d**) three-point bending device; (**e**) picture taken during the three-point bending process.

Specimens of 6 mm × 10 mm in size were made by wire-cutting. After grinding with 80~1500-grit sandpaper, followed by mechanical polishing, the specimens were etched with a 4% nitric acid–alcohol solution for 6~8 s, and then the corrosive solution was rinsed off from the specimens with alcohol. The representative microstructures before and after hot stamping were examined by optical microscopy (OM) and field-emission scanning electron microscopy (FE-SEM). According to the ASTM E-8 standard, tensile specimens with a gauge length of 50 mm and width of 12.5 mm were cut along the rolling direction (Figure 1a). All tensile specimens were tested on an MTS E45.105 machine at room temperature with a fixed crosshead speed of 3 mm/min. A 50 mm extensometer was equipped to measure the elongation of the gauge part (Figure 1c). When calculating the engineering stresses of the tensile tests, the precise thickness and width of the specimens’ gauge parts was measured one by one. The average strain of the 50 mm gauge part was applied for each specimen. For each sheet, three tests were performed, and the average values are reported in the later part of this article.

According to the VDA 238-100 standard, bending specimens with dimensions of 60 × 60 mm were prepared using EDM to ensure a smooth edge (Figure 1b). The three-point bending device is shown in Figure 1d. This device was installed on an MTS E45.105 tensile frame. Following the VDA 238-100 procedure, the roller gap was set to be twice the sheet thickness plus 0.5 mm, and the punch tip radius was 0.4 mm. The two supporting rollers were made of hardened tool steel and, thus, could be treated as rigid bodies and could rotate freely during the bending tests. The bending line was perpendicular to the sheet-rolling direction. Therefore, the principal stress was parallel to the sheet-rolling direction. During the tests, the punch moved downward at a velocity of 20 mm/min via a displacement control model until it touched the surface of the specimen, where a zero-bending load was registered. After the maximum bending load was achieved, the bending test was stopped when there was a load drop of 10 N from the previously reached load maximum. The entire punch load vs. displacement curve was recorded for each test, and the bending angle was calculated from the punch displacement using the formula provided in the VDA 238-100 standard.

### 2.2. Methodology to Characterize Fracture Strain under Plane-Strain Bending

**Figure 2:**
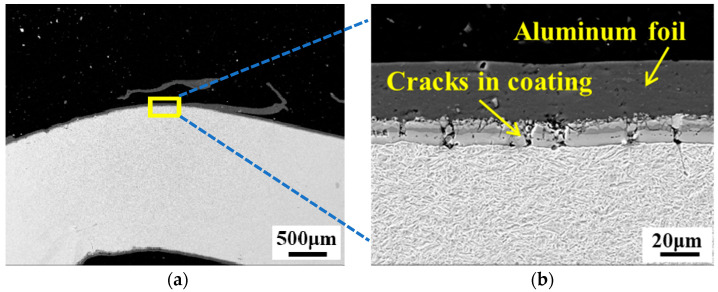
(**a**) SEM micrograph of the cross-section of a bent PHS specimen after heat treatment; (**b**) cracks in the Al–Si coating (the enlargement of the yellow rectangle in (**a**)).

Figure 2a shows the SEM micrograph of a bent PHS specimen at low magnification, while Figure 2b is a partial enlargement of Figure 2a. As clearly shown in Figure 2b, there were many cracks in the Al–Si coating layer. A previous study [13] measured the plane-strain fracture of PHS1500 via a tight-radius bending test using digital image correlation (DIC). It was also reported that Al–Si coatings crack at low strains, which can interfere with the DIC measurement. Therefore, the Al–Si coating on the PHS1500 had to be removed prior to testing. The method adopted in this study does not require the DIC technique; hence, the Al–Si coating was retained on all bending specimens.

To determine the actual fracture strains of the Al–Si-coated PHSs, a recently developed methodology was applied in this study [21]. The testing procedure of the method is detailed in this section.

Sheet metal is not uniformly stressed and strained along its thickness direction when bent. When bending strain and stress reach a threshold, cracks usually occur on the outer surface. For simplicity, when the punch is loaded (during bending) and unloaded (after removing the bending load), it is assumed that the inner and outer surfaces are circular. According to the bending theory, the equivalent strain due to plane-strain bending can be given as follows [21]:

(1) $$\bar{\varepsilon }=\frac{2}{\sqrt{3}}\cdot \ln\sqrt{\frac{R_{o}}{R_{i}}}$$

where $R_{o}$ and $R_{i}$ represent the radii of the outermost and innermost fibers, respectively, while $\bar{\varepsilon }$ is the equivalent strain of the outer fiber.

**Figure 3:**
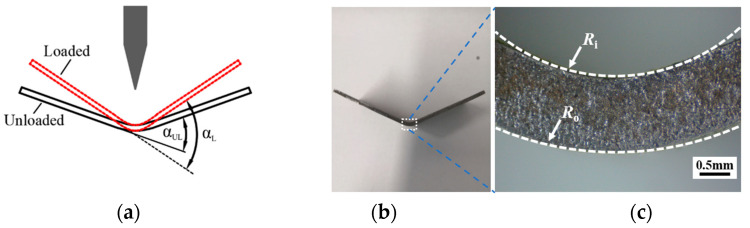
(**a**) Schematics of the cross-sections in the loaded and unloaded specimens; (**b**) cross-section of an unloaded specimen after being cut along the center line in the width direction; (**c**) enlarged view of the deformed zone (highlighted by the dashed line in Figure 1b) in an unloaded specimen.

After an elastoplastically bent sheet specimen is unloaded from its bent configuration, plastic deformation remains. This occurs when the shape of a loaded specimen (with a loaded bending angle, denoted as α_L_) changes to the unloaded configuration (with an unloaded bending angle, denoted as α_UL_), as schematically shown in Figure 3a. The elastoplastically deformed area is localized in a small area of the specimen when loaded (the rectangle outlined by dashed lines in Figure 3b) next to the punch–sheet contact zone, assuming that the innermost and outermost fibers of the deformed zone can be approximated as arcs in the unloaded configuration. According to another assumption, elastic strains are small compared to plastic strains, so they are ignored in this analysis. As a result, $\bar{\varepsilon }$ can be calculated by Equation (1) after the radii *R_i_* and *R_o_* (as shown in Figure 3c) are digitally determined using the curve-fitting function in AutoCAD 2020 software.

**Figure 4:**
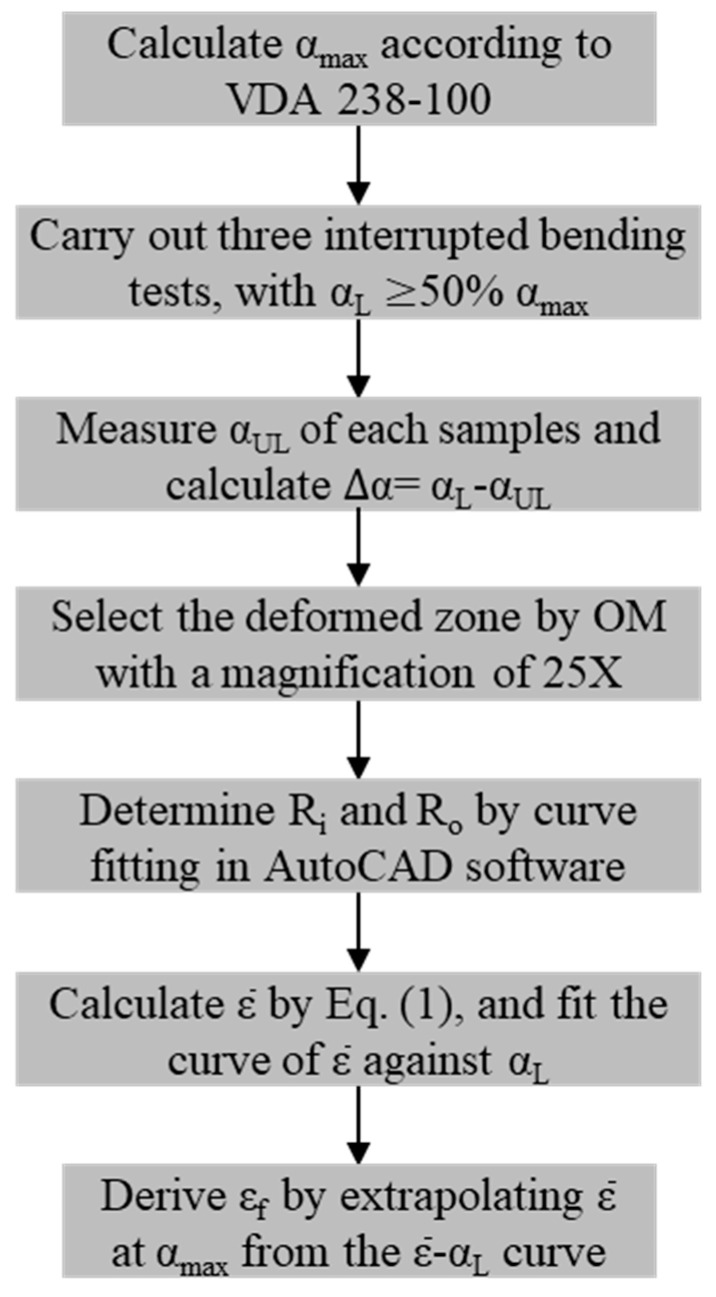
A flowchart describing the method used to determine the fracture strain ε_f_ under plane-strain bending.

Figure 4 illustrates the flowchart of the proposed method for determining the fracture strain of PHS under plane-strain bending, and a step-by-step procedure is provided below:

(1) Step one is to calculate the maximum bending angle α_max_ using the punch displacement at the peak punch load, according to the formulation given in the VDA 238-100 bending test procedure.
(2) Three interrupted bending tests are then carried out, each of which is stopped after reaching a prescribed punch displacement corresponding to a fraction (no less than 50%) of the α_max_. In this work, these stopping displacements were chosen such that bending angles of 30°, 40°, and 50° were achieved for Thick(_1.4/1.8_), while 35°, 45°, and 55° were chosen for Thin_(1.4/1.8)_.
(3) For each interrupted bending test, α_UL_ was directly measured on the unloaded specimen using AutoCAD software. The average springback angle Δα can be determined by comparing the loaded bending angle α_L_ (prescribed in the previous step) and the unloaded bending angle α_UL_.
(4) A deformed zone (marked with dashed lines in Figure 3b) in a cut specimen was selected as the area of interest. Then, an optical microscope (OM) with an appropriate magnification (25× in this study) was utilized to extract the profile.
(5) The profile image of the deformed zone was then imported into the AutoCAD software, and the radii of the innermost and outermost fibers were determined by curve fitting (*R_i_* and *R_o_* in Figure 3c).
(6) According to Equation (1), the equivalent strain $\bar{\varepsilon }$ at the outer surface for each interrupted bending test that was stopped at a prescribed α_L_ can be calculated.
(7) Finally, the fracture strain ε_f_ at the outer surface can be determined by extrapolating $\bar{\varepsilon }$ to the case when α_max_ is reached.

## 3. Results and Discussion

### 3.1. Microstructural Characterization

**Figure 5:**
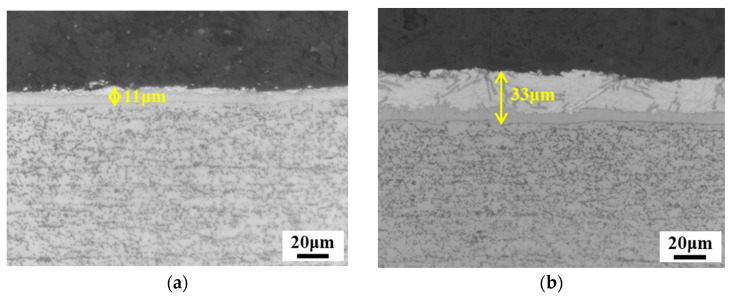
OM micrographs showing the coatings and microstructures before hot stamping: (**a**) Thin_1.4_; (**b**) Thick_1.4_.

The typical OM microstructures of the Thick_1.4_ and Thin_1.4_ PHS specimens before hot stamping are shown in Figure 5. The initial coating thickness of Thin_1.4_ PHS was around 11 μm, while the initial coating thickness of Thick_1.4_ PHS was about 33 μm. The as-received microstructure of both steels was ferrite plus pearlite. An interdiffusion layer of about 5–6 μm was observed for both steels, which grew during the hot stamping process when the steel was austenitized for a few minutes.

**Figure 6:**
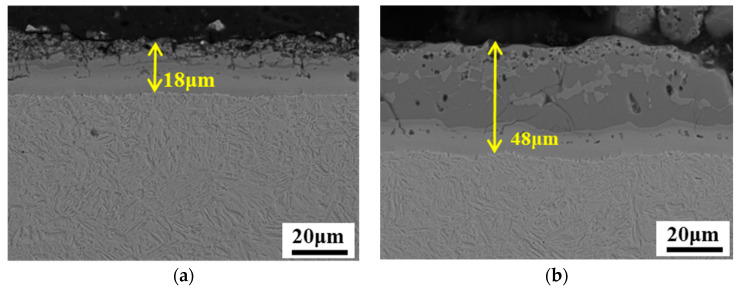
OM micrographs showing the coatings and microstructures After hot stamping: (**a**) Thin_1.4_; (**b**) Thick_1.4_.

Figure 6 exhibits the SEM microstructures of the Al–Si-coated PHSs after hot stamping. The matrix was mostly lath martensite, ensuring the 1500 MPa tensile strength and adequate fracture resistance. During austenitization, Al and Si atoms in the coating diffused into the steel substrate, while Fe atoms diffused from the steel substrate toward the Al–Si coating. As a result of this interdiffusion, the Al–Si coating grew as the austenitization temperature and time increased. The final coating thickness of Thin_1.4_ grew to about 18 μm, which was an increase of 7 μm compared to the original status. In comparison, the coating thickness of Thick_1.4_ increased from 33 μm to 48 μm. With the growth of the Al–Si coating, various Fe–Al intermetallic compounds were formed. Since carbon had almost zero solubility in the Fe–Al intermetallics, it was expelled to the steel substrate, leading to local enrichment near the coating–substrate interface. Subsequent quenching during the hot forming process hence produced martensite with high carbon content (higher than the nominal 0.22 wt.%). This martensite was more brittle than the lath martensite, and cracks were likely to appear early during the bending process. Therefore, local carbon enrichment at the interface of the Al–Si coating and the steel substrate is responsible for the reduced fracture resistance of the Al–Si-coated PHS. As previously proposed [6], a thin Al–Si coating is an effective method to alleviate the carbon enrichment and, hence, improve fracture resistance under plane-strain bending.

### 3.2. Mechanical Properties

The tensile properties of the Al–Si-coated steels are summarized in Table 2. It is noticeable that both the tensile strength and elongation of Thin_(1.4/1.8)_ are higher than those of Thick_(1.4/1.8)_. This can be plausibly explained as follows: The Al–Si coating after hot stamping consists of alternating alloy layers of FeAl_2_ and Fe_2_SiAl_2_, along with an interdiffusion layer that is mostly Al-enriched α-Fe. The FeAl_2_ and Fe_2_SiAl_2_ phases in the coating are hard and brittle, while the interdiffusion layer is soft and much more ductile. During the tensile test, the hard coating cannot withstand high load since it is full of microcracks (Figure 6). Therefore, the steel substrate effectively bears the external load. As shown in Figure 6, a thicker coating results in a reduced substrate thickness given that the total thickness of the coated steel is constant. In this case, a smaller ratio of the substrate to total thickness leads to lower tensile strength.

**Figure 7:**
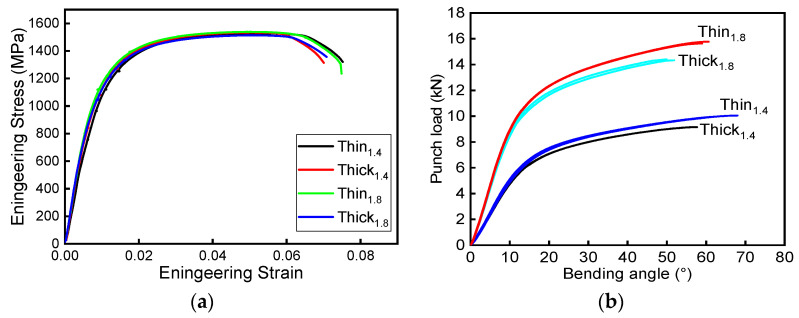
Mechanical properties of the four studied Al–Si-coated steels: (**a**) tensile properties; (**b**) bending results.

Three-point bending test results of the Al–Si-coated steels are shown in Figure 7b, in which punch load is plotted against the bending angle, which can be calculated from the punch displacement using the formulation given in the VDA 238-100 bending test procedure [18]. For the Thick_1.4_ and Thin_1.4_ specimens, the calculated maximum bending angles α_max_ corresponding to the maximum punch load are 66.1 ± 0.4° and 57.7 ± 0.2°, respectively, while the peak loads of Thick_1.4_ and Thin_1.4_ are 9.1 ± 0.1 kN and 10.0 ± 0.1 kN, respectively. Similarly, the α_max_ of Thick_1.8_ and Thin_1.8_ are 50.6 ± 0.5° and 59.7 ± 0.3°, respectively, while the peak loads are 14.4 ± 0.2 kN and 15.7 ± 0.1 kN, respectively. 

As shown in Table 2, the tensile strength of the thin Al–Si-coated steels is about 2% higher than that of the PHSs with regular Al–Si coating thickness. However, the average peak load of thin Al–Si-coated steel specimens increased by 8–9% compared to that of the PHSs with regular Al–Si coatings. In addition to the advantages of effective thickness, thin-coated materials also have a higher maximum bending angle. For the same material, an increase in the α_max_ brings greater resistance to fracture. At the same given bending angle, Thin_(1.4/1.8)_ showed higher punch loads than Thick_(1.4/1.8)_. One reason for this is that the average strength of thin materials is higher than that of thick materials, which lies in the whole thickness of the substrate involved in the deformation of thin materials being greater than that of thick materials. Secondly, according to the high bending angle, when fracture is about to happen, a greater fraction of thin materials is involved in deformation, leading to a higher peak load.

### 3.3. Determination of Fracture Strain

Based on the calculated bending angle α_max_ from Figure 7b, a series of interrupted bending tests were conducted for the four press-hardened steels with thin and thick Al–Si coatings. For each interrupted bending test case, the VDA test was stopped when a prescribed bending angle at loading (α_L_) was reached. The specimen was subsequently unloaded from the bending fixture, and the angle under unloaded conditions (α_UL_) was measured. The bending angles under loaded and unloaded conditions (i.e., α_L_ and α_UL_), along with the corresponding springback angle Δα (Δα = α_L_ − α_UL_), are summarized in Table 3 and Table 4. An increase in sheet thickness resulted in a slight decrease in the average Δα. As mentioned previously, Δα represents the elastic recovery of the material after unloading.

**Figure 8:**
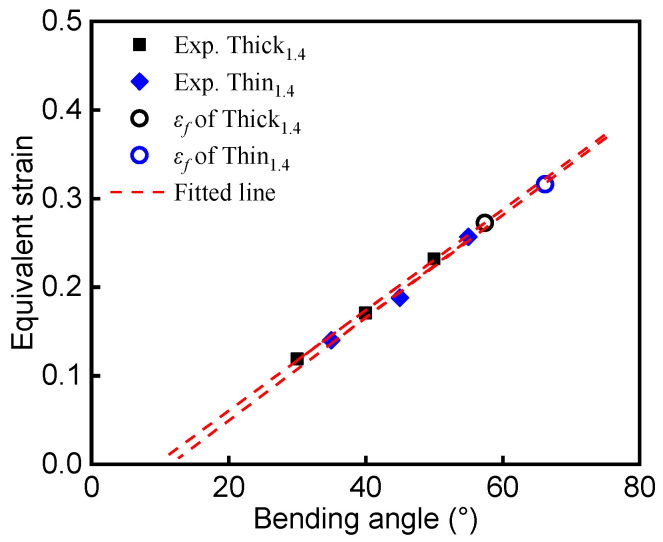
Equivalent strain calculated with the methodology as a function of the loaded bending angle α_L_ for the 1.4 mm Al–Si-coated steel.

**Figure 9:**
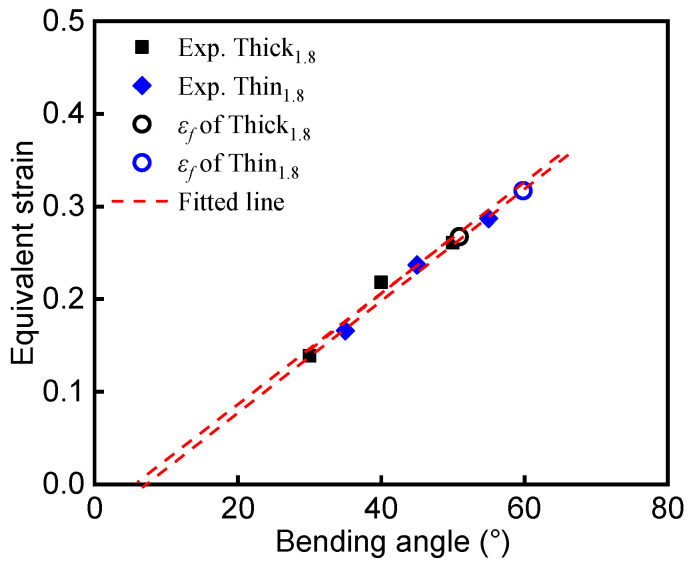
Equivalent strain calculated with the methodology as a function of the loaded bending angle α_L_ for the 1.8 mm Al–Si-coated steel.

Following the flowchart outlined in Figure 4, the deformed zone of the bent specimens was photographed using an OM at a low magnification of 25X. The OM profiles were imported to CAD software, and the radii *R_i_* and *R_o_* of the unloaded interrupted specimens were measured. In this study, since the elastic strain was negligibly small compared to the large plastic strain, the equivalent plastic strain was used to approximate the equivalent strain ($\bar{\varepsilon }$) of the loaded specimens. The equivalent plastic strain at the outer surface was calculated from the measured *R_i_* and *R_o_* of the unloaded interrupted specimens according to Equation (1). To determine the fracture strain ε_f_ of a sheet specimen under plane-strain bending, the equivalent strain data from the interrupted tests were fitted using a linear function, and the slope of the linear fitting was used for extrapolation of equivalent strain to the point of fracture, which corresponded to the calculated bending angle αmax. Finally, $\bar{\varepsilon }$ was plotted against the loaded bending angle α_L_ for the Al–Si-coated steels, as shown by the filled symbols in Figure 8 and Figure 9. The relationship between $\bar{\varepsilon }$ and the loaded bending angle was nearly linear. The dashed lines are the fitted lines for Thin_(1.4/1.8)_ and Thick_(1.4/1.8)_. In the initial stage of the bending process, only elastic deformation occurs. Referring to the curves in Figure 8 and Figure 9, $\bar{\varepsilon }$ equals zero when the fitting line intercepts at the *x*-axis (i.e., the interception is the amount of springback, Δα).

**Figure 10:**
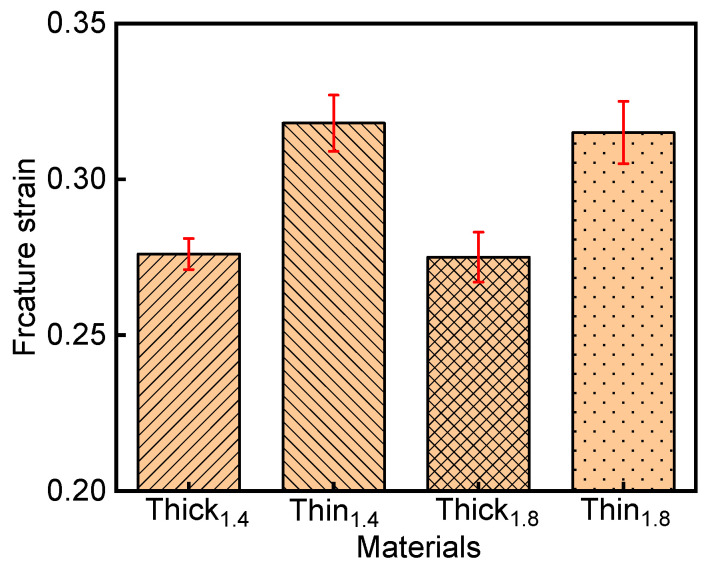
Fracture strain of the four Al–Si-coated steels.

The fracture strains of the Al–Si-coated steels, extrapolated from the fitting curves shown in Figure 8 and Figure 9, are summarized and plotted in Figure 10. The ε_f_ of Thin_1.4_ and Thick_1.4_ determined by our methodology was 0.318 ± 0.009 and 0.276 ± 0.005, respectively, while the ε_f_ of Thin_1.8_ and Thick_1.8_ was 0.315 ± 0.008 and 0.275 ± 0.010, respectively. As mentioned in the literature [4], fracture strain is independent of specimen thickness; hence, it is an appropriate material property for characterizing the fracture limits of press-hardened steels under plane-strain bending. Meanwhile, the ε_f_ results of Thick_(1.4/1.8)_ were comparable with results from the literature [8], where the major strain (i.e., fracture strain) measured by DIC was 0.27. Lastly, by reducing the thickness of the Al–Si coating, higher bending angle and fracture strain were achieved, directly supporting the mechanism of improving fracture resistance via surface carbon management [6].

### 3.4. The Critical Role of High Fracture Strain under Plane-Strain Bending

**Figure 11:**
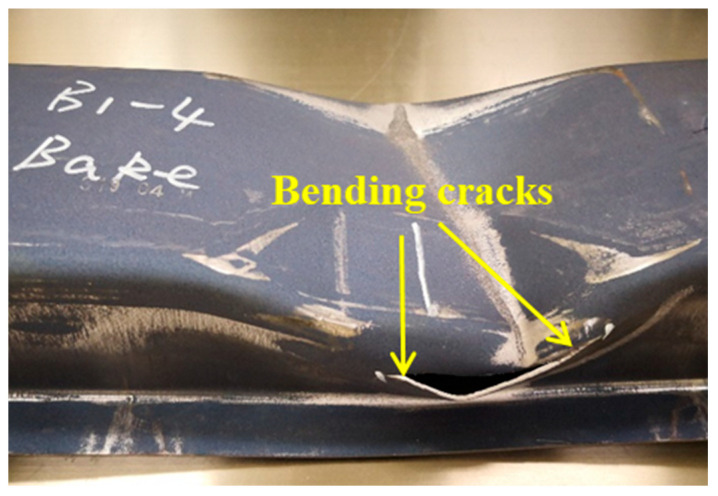
Cracks in a hot-stamped front bumper beam due to local bending after a static three-point bending test [7].

To ensure crash safety, adequate resistance to collision intrusion is needed for the body structural components of passenger vehicles. Crash-safety-related components are usually allowed to deform within a reasonable range and/or even undergo controlled fracture for achieving high energy absorption during a crash event. Given that most structural components are subjected to bending loads, as illustrated by the front bumper beam shown in Figure 11, favorable bendability is needed to manage crash energy. Yield strength, strain hardening, and tensile elongation are also important, because they collectively determine the occurrence of localized deformation, which usually precedes fracture [9].

Comparing the fracture strain of the steels, we concluded that a thinner Al–Si coating results in a higher fracture strain. Meanwhile, Figure 7 shows that the thinner-coated PHS has a higher punch load when bent to the same angle. As mentioned above, crash events usually occur under bending-dominated deformation, which is close to plane-strain conditions. Therefore, improving the fracture strain under plane strain is of significance to increasing vehicle safety [26].

**Figure 12:**
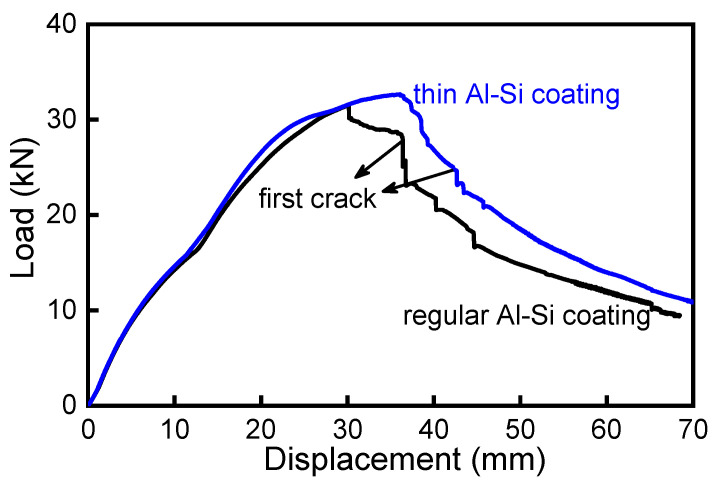
Performance comparison of press-hardened bumper beams in a static three-point bending test, with thin and regular Al–Si coatings [7].

In general, the elongation of a material is positively correlated with its work-hardening behavior. A higher work-hardening ability increases the uniform elongation and, hence, delays the occurrence of necking which, in turn, increases the total elongation. During a collision situation, the majority of the automobile components undergo bending-dominated deformation, and the potent areas of crack ignition are often close to plane-strain bending conditions. It was reported by Min et al. [11] that the strain instability occurs only when all of the material layers in the sheet have exceeded the forming limit strain. However, the through-thickness strain gradient of a bent specimen will suppress the material’s failure by necking. Instead, it is the stress and strain at the outer layer under the bending process that dictates the fracture behavior of materials. Therefore, better bendability (and, hence, higher fracture strain) of materials can directly contribute to component performance when bending is the relevant mode of deformation. As can be seen from Figure 12, the first crack occurred at a punch displacement of about 35 mm in the press-hardened bumper beam with a regular Al–Si coating thickness, while its counterpart beam with a thin Al–Si coating was able to delay the appearance of the first crack to 42 mm. In addition, it should be noted that the peak load was also higher (by about 1.3 kN) with the thin Al–Si-coated PHS bumper beam.

## 4. Conclusions

In this study, four Al–Si-coated 22MnB5 steels with different substrate and coating thicknesses were investigated. The following conclusions can be drawn:

(1) A recently developed methodology was employed to determine the fracture strain ε_f_ of Al–Si-coated 22MnB5 steels under plane-strain bending, which is difficult to measure by DIC methods due to the Al–Si coating cracking at low strains. Unlike the bending angle, the fracture strain is an intrinsic characteristic of the material and can be used directly as an input material parameter for computer-aided engineering (CAE) simulations.
(2) A thicker coating results in reduced substrate thickness. During tensile testing, a higher thickness ratio of thin-coated sheets was engaged in deformation, improving the total strength and elongation of the material simultaneously. At the same thickness, a larger bending angle represents higher fracture strain. The thickness of the substrate involved in deformation is also responsible for the peak load. In addition, greater toughness of the surface martensite leads to a higher bending angle.
(3) Materials with higher fracture strain have better collision energy absorption. For components and parts, thinner material design can be realized without sacrificing energy absorption ability.

## Figures and Tables

**Table 1:** Typical chemical composition of the Al–Si-coated 22MnB5 steels in this work (wt.%).

Materials	C	Mn	Si	Al	Ti	B
Thin_1.4_	0.22	1.21	0.23	0.034	0.029	0.004
Thick_1.4_	0.22	1.14	0.24	0.027	0.037	0.003
Thin_1.8_	0.22	1.20	0.24	0.058	0.029	0.003
Thick_1.8_	0.22	1.21	0.22	0.042	0.028	0.003

**Table 2:** Tensile properties of the Al–Si-coated steels after press-hardening.

Material	Yield Strength (MPa)	Tensile Strength (MPa)	Elongation (%)
Thin_1.4_	1227 ± 17	1518 ± 23	7.5 ± 0.4
Thick_1.4_	1189 ± 14	1489 ± 12	7.0 ± 0.5
Thin_1.8_	1219 ± 19	1525 ± 29	7.6 ± 0.3
Thick_1.8_	1205 ± 15	1502 ± 8	7.1 ± 0.2

**Table 3:** The results of interrupted bending tests for the 1.4 mm Al–Si-coated steels.

Thin_1.4_			Thick_1.4_		
α_L_(°)	α_UL_(°)	Δα(°)	α_L_(°)	α_UL_(°)	Δα(°)
35.0	25.5	9.6	30.1	21	9.1
45.0	35.7	9.3	40.6	31	9.6
55.0	47.3	7.7	50.0	41.5	8.5
Average Δα(°) 8.9 ± 1.0			Average Δα(°) 9.1 ± 0.6		

**Table 4:** The results of interrupted bending tests for the 1.8 mm Al–Si-coated steels.

Thin_1.8_			Thick_1.8_		
α_L_(°)	α_UL_(°)	Δα(°)	α_L_(°)	α_UL_(°)	Δα(°)
35.0	26.3	9.6	30.1	21	9.1
45.0	36.8	9.3	40.6	31	9.6
55.0	46.9	7.7	50.0	41.5	8.5
Average Δα(°) 8.3 ± 0.3			Average Δα(°) 8.3 ± 0.5		

## Data Availability

Not applicable.

## References

[1] Liu L., Yu Q., Wang Z., Ell J., Huang M.X., Ritchie R.O. (2020). Making ultrastrong steel tough by grain-boundary delamination. Science.

[2] Abio A., Bonada F., Pujante J., Grané M., Nievas N., Lange D., Pujol O. (2022). Machine Learning-Based Surrogate Model for Press Hardening Process of 22MnB5 Sheet Steel Simulation in Industry 4.0. Materials.

[3] Tong C., Rong Q., Yardley V., Li X., Luo J., Zhu G., Shi Z. (2020). New Developments and Future Trends in Low-Temperature Hot Stamping Technologies: A Review. Metals.

[4] Löbbe C., Hering O., Hiegemann L., Tekkaya A.E. (2016). Setting Mechanical Properties of High Strength Steels for Rapid Hot Forming Processes. Materials.

[5] Wróbel I., Skowronek A., Grajcar A. (2022). A Review on Hot Stamping of Advanced High-Strength Steels: Technological-Metallurgical Aspects and Numerical Simulation. Symmetry.

[6] Jin X., Gong Y., Han X., Du H., Ding W., Zhu B., Zhang Y., Feng Y., Ma M., Liang B. (2020). A Review of Current State and Prospect of the Manufacturing and Application of Advanced Hot Stamping Automobile Steels. Acta Metall. Sin..

[7] Yi H., Chang Z., Cai H., Du P., Yang D. (2020). Strength, ductility and fracture strain of press-hardening steels. Acta Metall. Sin..

[8] Hou Z., Min J., Wang J., Lu Q., He Z., Chai Z., Xu W. (2021). Effect of Rapid Heating on Microstructure and Tensile Properties of a Novel Coating-Free Oxidation-Resistant Press-Hardening Steel. JOM.

[9] Yoo J., Kim S., Jo M., Kim S., Oh J., Kim S., Lee S., Sohn S. (2022). Effects of Al-Si coating structures on bendability and resistance to hydrogen embrittlement in 1.5-GPa-grade hot-press-forming steel. Acta Mater..

[10] Thom V., Duc H., Nguyen C., Nguyen D. (2022). Thermal Buckling Analysis of Cracked Functionally Graded Plates. Int. J. Struct. Stab. Dyn..

[11] Cong P., Van T., Duc H. (2022). Phase field model for fracture based on modified couple stress. Eng. Fract. Mech..

[12] Nam V., Duc H., Nguyen C., Thom V., Hong T. (2019). Phase-field buckling analysis of cracked stiffened functionally graded plates. Compos. Struct..

[13] Cheong K., Butcher C., Dykeman J. (2018). The Influence of the Through-Thickness Strain Gradients on the Fracture Characterization of Advanced High-Strength Steels. SAE Int. J. Mater. Manuf..

[14] Rosenthal S., Maaß F., Kamaliev M., Hahn M., Gies S., Tekkaya A.E. (2020). Lightweight in Automotive Components by Forming Technology. Automot. Innov..

[15] Wagner L., Larour P., Dolzer D., Leomann F., Suppan C. (2020). Experimental issues in the instrumented 3 point bending VDA238-100 test. IOP Conf. Ser. Mater. Sci. Eng..

[16] Tang B., Wu F., Guo N., Liu J., Ge H., Bruschi S., Li X. (2020). Numerical modeling of ductile fracture of hot stamped 22MnB5 boron steel parts in three-point bending. Int. J. Mech. Sci..

[17] Cheong K., Omer K., Butcher C., George R., Dykeman J. (2017). Evaluation of the VDA 238-100 tight radius bending test using digital image correlation strain measurement. IOP Publ. J. Phys. Conf. Ser..

[18] Verband der Automobilindustrie (VDA) (2010). VDA 238-100 Plate Bending Test for Metallic Materials.

[19] Min J., Stoughton T.B., Carsley J.E., Lin J. (2016). Compensation for process-dependent effects in the determination of localized necking limits. Int. J. Mech. Sci..

[20] Noder J., Abedini A., Butcher C. (2020). Evaluation of the VDA 238–100 Tight Radius Bend Test for Plane Strain Fracture Characterization of Automotive Sheet Metals. Exp. Mech..

[21] Cai H.L., Wang J.F., Wu D., Yi H.L. (2021). A Simple Methodology to Determine Fracture Strain of Press-Hardened Steels Under Plane Strain Bending. Metall. Mater. Trans. A.

[22] Noder J., Abedini A., Butcher C. (2021). New Methodologies for Fracture Detection of Automotive Steels in Tight Radius Bending: Application to the VDA 238–100 V-Bend Test. Exp. Mech..

[23] Drillet P., Spehner D., Kefferstein R. (2017). Coated Steel Strips, Methods of Making the Same, Methods of Using the Same, Stamping Blanks Pre-Pared from the Same, Stamped Products Prepared from the Same, and Articles of Manufacture Which Contain Such a Stamped Product. China Patent.

[24] Cho L., Golem L., Seo E., Bhattacharya D., Speer J., Findley K. (2020). Microstructural characteristics and mechanical properties of the Al–Si coating on press hardened 22MnB5 steel. J. Alloys Compd..

[25] Yi H., Liu H., Chang Z., Xiong X. (2020). Hot Stamped Component, Pre-Coated Steel Sheet Used for Hot Stamping and Hot Stamping Process. China Patent.

[26] Niu G., Zurob H., Misra R.D.K., Tang Q., Zhang Z., Nguyen M., Wang L., Wu H., Zou Y. (2022). Superior fracture toughness in a high-strength austenitic steel with heterogeneous lamellar microstructure. Acta Mater..

